# Strategy and cost analysis of vascular access for hemodialysis in North Macedonia

**DOI:** 10.1590/1677-5449.202500292

**Published:** 2026-01-12

**Authors:** Nikola Gjorgjievski, Vlatko Karanfiovski, Petar Dejanov, Igor Nikolov

**Affiliations:** 1 University Clinic of Nephrology, Faculty of Medicine, Ss. Cyril and Methodius University, Skopje, North Macedonia.

**Keywords:** vascular access, hemodialysis, arteriovenous fistula, catheter, budget, acesso vascular, hemodiálise, fístula arteriovenosa, cateter, orçamento

## Abstract

**Background:**

Vascular access (VA) is a critical component of hemodialysis (HD). Arteriovenous fistulas (AVFs) are associated with lower morbidity and mortality compared to central venous catheters (CVCs). However, VA practice varies across different healthcare systems.

**Objectives:**

The aim of our study was to evaluate the trends, costs, and distribution of VA types used in North Macedonia over two decades.

**Methods:**

This retrospective study reviewed VA procedures performed at the University Clinic of Nephrology in Skopje from 2002 to 2023, highlighting trends and financial implications. Data on AVFs, tunneled central catheters (TCCs), and temporary catheters were collected and analyzed. Preoperative Doppler ultrasound assessments and follow-up evaluations were used to monitor AVF maturation and CVC placement. The cost analysis was based on the Diagnostic-Related Group coding system.

**Results:**

A total of 25 532 VA procedures were performed, including 5798 AVFs (83% of permanent VA), 1199 TCCs (17%), and 18 535 temporary CVCs. The number of AVFs steadily increased during the whole of the analyzed period, except in 2020 due to the COVID-19 pandemic. Temporary femoral vein catheterization accounted for 90% of CVCs. The financial burden of VA increased from 6.6% of the clinic’s budget in 2018 to 9.3% in 2022, with a notable rise in CVC-related expenses.

**Conclusions:**

In North Macedonia, use of temporary CVCs at dialysis initiation remains high. Increasing the number of preemptive AVFs and improving long-term VA planning are essential to optimize patient outcomes and reduce healthcare costs.

## Resumo

**Contexto:**

O acesso vascular (AV) é um componente fundamental da hemodiálise (HD). As fístulas arteriovenosas (FAVs) estão associadas a menor morbidade e mortalidade quando comparadas aos cateteres venosos centrais (CVCs). No entanto, a prática de AV varia entre diferentes sistemas de saúde.

**Objetivos:**

O objetivo deste estudo foi avaliar as tendências, os custos e a distribuição dos tipos de AV utilizados na Macedônia do Norte ao longo de duas décadas.

**Métodos:**

Este estudo retrospectivo revisou os procedimentos de AV realizados na Clínica Universitária de Nefrologia, em Skopje, de 2002 a 2023, destacando tendências e implicações financeiras. Dados sobre FAVs, cateteres centrais tunelizados (CCTs) e cateteres temporários foram coletados e analisados. O monitoramento da maturação das FAVs e da colocação dos CVCs foi realizado por meio de avaliações pré-operatórias com ultrassom Doppler e acompanhamento. A análise de custos baseou-se no sistema de codificação *Diagnostic-Related Group*.

**Resultados:**

Foram realizados 25.532 procedimentos de AV, incluindo 5.798 FAVs (83% dos AVs permanentes), 1.199 CCTs (17%) e 18.535 CVCs temporários. O número de FAVs aumentou gradativamente ao longo do período analisado, exceto em 2020, devido à pandemia de covid-19. A cateterização temporária da veia femoral representou 90% dos CVCs. O impacto financeiro dos AVs passou de 6,6% do orçamento da clínica em 2018 para 9,3% em 2022, com aumento expressivo nas despesas relacionadas aos CVCs.

**Conclusões:**

Na Macedônia do Norte, o uso de CVCs temporários no início da diálise permanece elevado. Aumentar o número de FAVs preventivas e melhorar o planejamento a longo prazo dos AVs são ações essenciais para otimizar os resultados dos pacientes e reduzir os custos com saúde.

## INTRODUCTION

Chronic kidney disease is one of the fastest-growing diseases worldwide. Kidney transplantation (KTx), hemodialysis (HD), and peritoneal dialysis (PD) are treatment modalities for patients with end-stage chronic kidney disease (ESKD).^1^ Insufficient access for patients to kidney replacement therapy (KRT) results in premature death, which was estimated to be the case for more than 2 million people globally in 2010.^2^ This burden may be further compounded by shortages of organs for transplantation and inadequate vascular access (VA) for HD, particularly in low-income and middle-income countries.^2^ The pivotal element in delivery of adequate HD is successful creation of VA: arteriovenous fistulas (AVFs), arteriovenous grafts (AVGs), and temporary and permanent central venous catheters (CVCs).^3^ The "fistula first" policy is based on evidence from large observational studies showing that AVFs are the best option for patients who require HD, due to a lower risk of death from infection and cardiovascular diseases compared to CVCs.^4^ However, the proportion of elderly HD patients suffering from diabetes mellitus and cardiovascular diseases is increasing worldwide and use of CVCs has become more frequent due to unsuccessful maturation of AVFs in these patients. Providing the best type of VA is multidisciplinary and depends on different factors including age, primary kidney disease, life expectancy, complication rates, availability of surgeons, radiologists, and nephrologists, availability of catheters/materials, indication or urgency for dialysis, native vascular anatomy, and provider preferences.^4^ The Kidney Disease Outcomes Quality Initiative (KDOQI) vascular access guidelines strongly encourage clinicians to maximize AVF placement and determine the optimal VA for each patient, because there is a large benefit in an overall a reduction in the number of procedures and costs.^3^ The approach to VA has evolved dramatically over the past few years. The optimal proportion of initial VA placements will differ at different dialysis centers, depending on the relative patient age and burden of cardiovascular comorbidities. From the patients’ perspective, there is no right or wrong method of access; the best VA is the right access, in the right patients, at the right time, for the right reason.^5^

## MATERIALS AND METHODS

This study included patients requiring HD who underwent VA procedures performed by nephrologists between 2002 and 2023. All nephrologists in our clinic are trained in temporary femoral central vein catheterization for urgent HD, while four specialized nephrologists perform temporary CVC placements in the jugular and subclavian veins, as well as creation of permanent VA.

Preoperative assessment of upper limb arteries and veins using Doppler ultrasound (DUS) was conducted for all patients undergoing AVF creation, ensuring optimal vessel selection and procedural success. Additionally, AVF maturation was monitored through follow-up DUS examinations. DUS was also the standard technique for guiding placement of CVCs in the jugular vein. Data were collected on the types and frequency of VA procedures performed, including AVFs, permanent CVCs, and temporary CVCs. Descriptive statistics were used to analyze trends in VA utilization, with annual assessments of the number and proportion of each access type. The financial impact of VA procedures was evaluated over time, incorporating hospitalization costs and changes in distribution of access types based on the Diagnostic-Related Group (DRG) coding system. A comparative analysis was conducted to assess shifts in VA utilization patterns before and after the COVID-19 pandemic. We adhered to the CHEERS-Consolidated Health Economic Evaluation Reporting Standards guidance (Husereau et al.)^6^ to ensure comprehensive and transparent reporting of our methods and findings. This research was approved by the Local Ethics Committee and conducted in accordance with the International Conference on Harmonization-Good Clinical Practice (ICH-GCP) guidelines and the Declaration of Helsinki to ensure adherence to ethical and clinical standards.

## RESULTS

**Figure 1:**
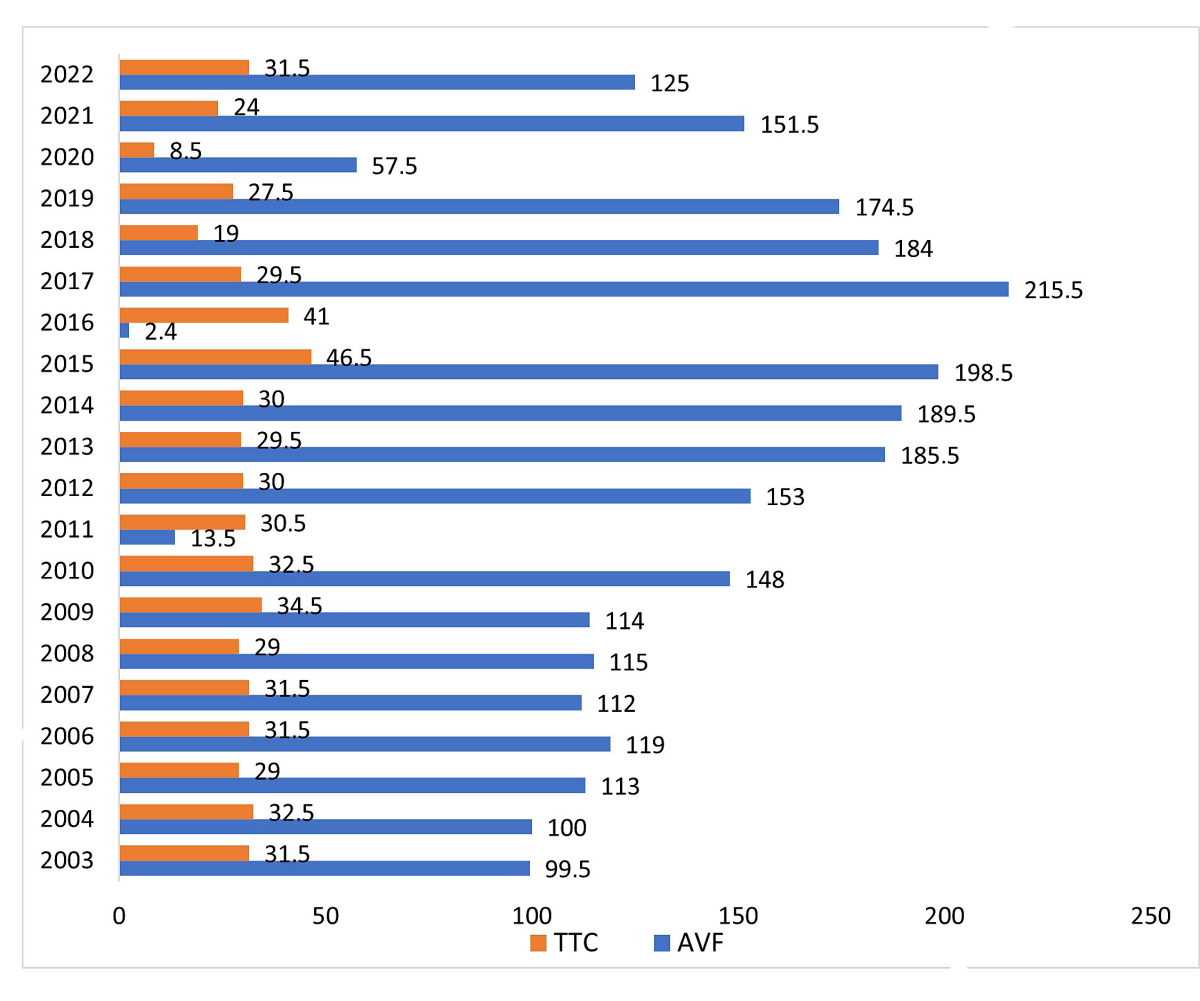
The number (pmp) of permanent vascular accesses created for HD per year, 2003-2022. TCC: Tunneled central catheters; AVF: Arteriovenous fistulas.

The Department for Vascular Access at the University Clinic of Nephrology in Skopje provides around 85% of all VA created in North Macedonia. The department was established four decades ago and currently the fifth generation of nephrologists specialized in the field of VA are active in creating temporary and permanent accesses for HD.^7,8^ In the period from 2002 until 2023, clinicians at our clinic created 25 532 different vascular hemodialysis accesses. In addition, the number of permanent VA created was 6997or 27.4% of all VA created, 5798 (83%) of which were AVFs and 1199 (17%) of which were tunneled central catheters (TCCs). The number of temporary VA created was 18 535 or 72.5%, 16 682 (90.0%) of which were femoral vein catheters (FVCs), 787 (4.2%) of which were jugular vein catheters (JVCs), and 1066 (5.75%) of which were subclavian vein catheters (SVCs). The number of vascular grafts created was insignificantly low. The number of AVFs created has been continuously growing from 2002 to 2018, peaking in 2017, when 215.5 per million people (pmp) were created, but in 2020 the number decreased sharply due to the COVID-19 pandemic (Figure 1). Also, the number of TCCs varied slightly over the years, with an average of 60 per year (30 pmp) (Figure 1). According to the CHEERS guidelines, these trends highlight the importance of assessing the long-term economic impacts of such changes in access types. We conducted an economic analysis incorporating the direct medical costs of both VA procedures and hospitalization, reflecting our adherence to the CHEERS recommendations for transparency in reporting and comprehensive economic analysis.^6^ This study adopts a healthcare system perspective, analyzing the direct medical costs associated with VA procedures.

Regarding the costs, the analyses were performed according to the DRG coding system recommended by the Health Insurance Fund of North Macedonia. DRG code L09C was used for permanent VA, which costs 538 US Dollars per hospitalization, and DRG code Z64B was used for temporary VA, which costs 182 US Dollars per hospitalization.^9^Table 1 presents all charges for VA hospitalization at our clinic during the last five years. Our clinic has a total budget of 3 868 161 US Dollars, 6.6% of which were spent to create VA in 2018, rising to 9.3% in 2022. Compared to 2018, costs related to VA (temporary and permanent) had increased by 28.2% in 2023, and the greatest increase, of 56.5%, was observed for CVC costs.

**Table 1:** Charges for vascular access hospitalization in our clinic in the last 5 years.

**Year**	**Total cost of permanent VA for HD (AVFs or TCCs) L09 = 538 US$ per hospitalization**		**Total cost of temporary VA for HD (CVCs) Z64B=182US$ per hospitalization**		**Total cost of all VA for HD (AVFs, TTCs, and CVCs) compared to the Clinic’s budget.(Total budget is 3 868 181 US$)**
	**Number of hospitalizations**	**Cost of hospitalization US$**	**Number of hospitalizations**	**Cost of hospitalization US$**	**Total spend for hospitalizations US$**
**2018**	388	208 744	262	47 684	256 428 (6.6%)
**2019**	412	221 656	327	41 856	263 512 ( 6.8%)
**2020**	Covid	Covid	Covid	Covid	Covid
**2021**	356	191 528	550	100 100	291 628 (7.5%)
**2022**	462	248 556	560	101 920	350 476 (9.1%)
**2023**	460	247 480	602	109 564	357 044 (9.3%)

VA: Vascular access; AVFs: Arteriovenous fistulas; TCCs: Tunneled central catheters; HD: Hemodialysis; CVCs: Central venous catheters; US$: United States dollars; MKD: Macedonian denars.

The average value of 1 US$ in Macedonian denars (MKD) in each year was: 2018 1US$=52.98MKD; 2019 1US$=54.95MKD;

2020 1US$=54.24MKD; 2021 1US$=52.12MKD; 2022 1US$=58.61MKD; 2023 1US$=56.96MKD.

This aligns with CHEERS guidelines’ focus on evaluating the cost-effectiveness of various healthcare interventions, considering both the direct costs and the long-term economic burden on the healthcare system.

## DISCUSSION

In 2023, an electronic survey of 167 countries in the world focused on types of accesses used to initiate HD, showing that 31 countries (25% of surveyed countries) had >75% of patients initiating dialysis with temporary CVCs, and that the rate was higher in low-income countries - LICs (57% of all LICs) than in countries in other income categories: lower-middle-income countries - LMICs (21%), upper-middle-income countries - UMICs (23%), and higher-income countries - HICs (5%).^10^ Although North Macedonia is a UMIC, more than 90% of all patients start HD with a CVC; the number of preemptive AVFs created is insignificantly small (only 10-15 AVFs per year). Our study demonstrates a persistently high rate of use of temporary CVCs at the initiation of HD in North Macedonia, with only a modest increase in AVFs over the study period. These findings highlight challenges in VA planning that are critical when considering the KDOQI guidance.^3^ The guidelines strongly recommend an AVF-first approach, emphasizing that AVFs should be placed in as many patients as possible due to their lower rates of infection, thrombosis, and mortality compared to CVCs. Compared with the period from 1976 to 1999, during the analyzed period from 2002 to 2023, we tripled the number of all interventions related to HD vascular accesses performed at our department: the number of temporary and permanent catheters increased by 75% (4964 vs 19 734) and the number of AVFs created increased by 47% (3114 vs 5798).^7,8^ This situation could be explained by the increase in the total number of HD patients and increases in expertise, technical support, and the number of nephrologists.^7^ The number of temporary FVCs grew constantly during the period from 2002 to 2023 and the largest number of FVCs placed was in 2020 (654.5 pmp), due to the COVID-19 pandemic. Also, the number of AVFs created decreased, and many of the temporary FVCs were used as access for prolonged ambulatory HD. However, placement of JVCs under ultrasound control started in 2008 at our clinic and since then their number has been constantly increasing, while the number of SVCs showed a decreasing trend. Our previous results showed that FVCs had a lower rate of early complications, with equal or lower rates of stenosis, thrombosis, and infections compared to subclavian and jugular vein catheterizations.^7,8^ Moreover, FVCs do not interfere with the creation of an AVF in the upper limbs, which is a favorable aspect of FVCs as a first option for temporary VA. Also, FVCs can be used without any problem for a longer period for ambulatory HD under permanent care from a team specially trained in vascular access.^5,7,8^ However, the situation is different among patients after the start of HD; almost 80% of our HD patients use AVFs, around 14% of patients use TCCs, and the remainder use temporary CVCs. The KDOQI guidelines recommend limiting use of TCCs to cases in which AVFs or grafts are not feasible. In the future, we can move closer to achieving the ideal KDOQI targets of >60% AVF use at dialysis initiation and <10% catheter dependence.^3^

The number of patients who use arteriovenous grafts is <1% due to the insufficient experience and lack of tradition of creating arteriovenous grafts at our institution.^7^ On the other hand, the Dialysis Outcomes and Practice Patterns Study (DOPPS) showed that the great majority of Japanese HD patients (91%) dialyze with an AVF, versus 68% in the United States. AVGs accounted for 12% to 13% of all accesses created in the DOPPS phases 4 to 5 in Europe, Australia and New Zealand, and Japan, versus 25% in the United States.^11^

At our clinic, the number of hospitalizations associated with VA for HD constitutes a significant proportion of the total number of hospitalizations, and in 2018 they accounted for 30.28% (18.08% related to permanent VA for HD and 12.2% to temporary VA for HD). This percentage includes patients in whom CVCs were placed for urgent HD, change of CVCs due to malfunction and/or infection, patients in need of temporary access due to malfunction of permanent access for HD, as well as patients in whom AVFs were created or TCCs were placed. CVCs were placed on an outpatient basis, while the average duration of hospitalization in patients with permanent VA was 1 day.

Over the last 5 years, the total number of VA-related hospitalizations showed a continuous increase and this growth was mainly due to an increase in the total number of CVCs placed, and less to an increase in the number of AVFs and TCCs created (Table 1). This situation might be due to an increase in the total number of HD patients, an increase in the number of patients who presented with the need for urgent HD without previous nephrological monitoring, as well as a change in the CVC provider. Our outpatient or short-term hospitalization approach has proven to be a successful model and Mishler R et al. conducted a retrospective analysis of more than 295 000 HD patients, concluding that the development of an outpatient VA center was associated with a significant decrease in VA-related hospitalization and missed outpatient dialysis treatments.^12^

The KDOQI guidelines emphasize AVF-first strategies as a cost-effective approach, which aligns with the CHEERS recommendation to assess long-term economic impact rather than short-term procedural costs.^3,6^ Our economic evaluation highlights that even small changes in unit costs can have a notable impact on the overall financial burden. This finding emphasizes the importance of cost-effective strategies in managing hemodialysis access. Nonetheless, adherence to CHEERS guidelines ensures that our findings are reported with a high degree of transparency and reproducibility.^6^

Our study has several limitations. First, the study is a retrospective analysis based on single-center experience. Second, it does not include detailed analyses of patient outcomes related to different VA, such as infection rates, thrombosis, long-term patency, and patient survival, which are critical for evaluating the effectiveness of different VA strategies. Also, the study provides an estimate of the cost using the DRG coding system, it does not account for indirect costs, such as complications related to different VA types, readmissions, or the long-term economic burden of repeated CVC-related infections, which are limitations according to the CHEERS guidelines.^6^

These limitations highlight areas for future research, such as prospective studies on patient outcomes, cost-effectiveness analyses, and strategies to improve early nephrology referral and preemptive AVF creation.

## CONCLUSION

This study highlights the evolving landscape of VA for HD in North Macedonia over two decades and more than 25 000 patients. Despite increasing adoption of AVFs among long-term dialysis patients, the high prevalence of temporary CVCs at dialysis initiation remains a challenge. While advances in surgical expertise and preoperative assessments have contributed to improved AVF utilization, the persistent reliance on temporary CVCs underscores a need for earlier nephrology referrals and preemptive AVF creation. Additionally, the rising financial burden associated with VA, particularly the increasing costs of CVC-related procedures, reinforces the importance of cost-effective strategies that prioritize AVF placement. Moving forward, a multidisciplinary approach focusing on patient education, timely vascular access planning, and healthcare policy improvements is essential to enhancing patient outcomes and reducing overall healthcare expenditure.

### Statement

The article is in accordance with the International Conference of Harmonization-Good Clinical Practice (ICH-GCP) requirements, national laws, and the declaration of Helsinki, and was approved by the Local Ethics Committee. Written informed consent was obtained from all authors.

## Data Availability

Data included in the article/supplemental material: “All data generated or analyzed are included in this article and/or in the supplemental material.”
